# Hypothalamus-Olfactory System Crosstalk: Orexin A Immunostaining in Mice

**DOI:** 10.3389/fnana.2012.00044

**Published:** 2012-11-08

**Authors:** Jean Gascuel, Aleth Lemoine, Caroline Rigault, Frédérique Datiche, Alexandre Benani, Luc Penicaud, Laura Lopez-Mascaraque

**Affiliations:** ^1^Instituto Cajal, CSIC, Avda del Doctor ArceMadrid, Spain; ^2^CNRS UMR 6265, Centre des Sciences du Goût et de l’AlimentationDijon, France; ^3^Institut National de la Recherche Agronomique UMR 1324, Centre des Sciences du Goût et de l’AlimentationDijon, France; ^4^Université de Bourgogne UMR CSGA, Centre des Sciences du Goût et de l’AlimentationDijon, France

**Keywords:** orexin A, olfactory system, hypothalamus, food intake behavior, AOB, MOB, immunocytology

## Abstract

It is well known that olfaction influences food intake, and conversely, that an individual’s nutritional status modulates olfactory sensitivity. However, what is still poorly understood is the neuronal correlate of this relationship, as well as the connections between the olfactory bulb and the hypothalamus. The goal of this report is to analyze the relationship between the olfactory bulb and hypothalamus, focusing on orexin A immunostaining, a hypothalamic neuropeptide that is thought to play a role in states of sleep/wakefulness. Interestingly, orexin A has also been described as a food intake stimulator. Such an effect may be due in part to the stimulation of the olfactory bulbar pathway. In rats, orexin positive cells are concentrated strictly in the lateral hypothalamus, while their projections invade nearly the entire brain including the olfactory system. Therefore, orexin appears to be a good candidate to play a pivotal role in connecting olfactory and hypothalamic pathways. So far, orexin has been described in rats, however, there is still a lack of information concerning its expression in the brains of adult and developing mice. In this context, we revisited the orexin A pattern in adult and developing mice using immunohistological methods and confocal microscopy. Besides minor differences, orexin A immunostaining in mice shares many features with those observed in rats. In the olfactory bulb, even though there are few orexin projections, they reach all the different layers of the olfactory bulb. In contrast to the presence of orexin projections in the main olfactory bulb, almost none have been found in the accessory olfactory bulb. The developmental expression of orexin A supports the hypothesis that orexin expression only appears post-natally.

## Introduction

Everyone at some point in their lives has been astonished by the influence that feeding has on olfactory sensitivity, particularly in the perception of food-associated odorants. This suggests that one of the first ways in which the brain regulates food intake behavior is by modulating the perception of the food odorant itself. Indeed, such change in sensory perception has been defined as alliesthesia (Cabanac and Duclaux, 1973; Duclaux et al., 1973) and sensory specific satiety (SSS; Yeomans, 2006). At the most peripheral level – the olfactory epithelium (OE) – many neuropeptides and metabolic hormones such as Gonadotropin-Releasing Hormone (GnRH), Neuronal Peptide Y (NPY), leptin, adiponectin, and orexins are thought to modulate the sensitivity of olfactory sensory neurons in different species. For instance, in lower vertebrates, LHRH/GnRH, released by the nervus terminalis (NT) at the OE level (Wirsig-Wiechmann, 1993; Oka, 1997), modulates olfactory neuronal sensitivity (Kawai et al., 2009) reducing evoked responses to food odorant cues during the reproductive period in Axolotl. This allows the olfactory system to be available predominantly for odorants involved in mating (Mousley et al., 2006). In contrast, NPY, an orexigenic neuropeptide, is also released producing an inverse effect in the OE, i.e., causing an increase in sensitivity to food odorants (Mousley et al., 2006). In mammals, hormonal mechanisms have also been shown to modulate the sensitivity of the olfactory system. Leptin – a hormone produced by adipocytes in proportion to the fat content, and involved in modulation of the neuronal network linked to energy balance (Friedman, 2002; Pinto et al., 2004) – is present in the OE and has a modulatory effect on olfactory sensory neurons (Savigner et al., 2009). Similarly, adiponectin receptors 1 (receptor for adiponectin, a hormone involved in glucose and lipid metabolism) are expressed by mature sensory neurons (Hass et al., 2008), suggesting that this peptide is also able to modulate olfactory responses.

**Figure 1:**
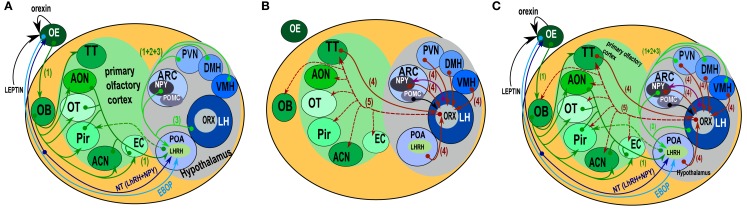
**Schematic representation of the “connectome” between the olfactory system and the hypothalamus**. **(A)** Paracrine, extrabulbar connection, and afferents to LHRH/GnRH hypothalamic neurons. Orexin and leptin could modulate olfactory neuron sensitivity by paracrine action. The EBOP (Extra Bulbar Olfactory Pathway) is made up of sensory neurons that project directly to the POA in the hypothalamus. However, this pathway has only been identified in lower vertebrates. In contrast, the Nervus Terminalis is known in mammals and humans. This complex structure, which projects both to the olfactory epithelium and the POA, is thought to play a bidirectional modulatory role. LHRH/GnRH neurons get afferents from nearly all the areas of the olfactory system (including the OB), and from the most important hypothalamic structures involved in the regulation of food intake behavior (1, 2). However, no direct output onto olfactory structures has been evidenced. **(B)** Afferents onto orexin neurons (4). Orexin neurons received input from nearly all of the hypothalamic areas. Interestingly, they also received input from the Tenia Tecta (TT) of the olfactory cortex. From the arcuate nucleus, both POMC and NPY (6) neurons project onto orexin neurons. Both the input and output (Peyron et al., 1998) of orexin neurons are represented. Output data are only from rats, but they provided evidence of a putative output from orexin neurons onto the olfactory structures and input from the TT of the olfactory cortex onto orexin neurons. **(C)** This synthetic representation, even though incomplete, reveals the complexity of the putative interactions between olfaction and hypothalamic areas. (1 = Yoon et al., 2005; 2 = Boehm et al., 2005; 3, 6 = Yoshida et al., 2006; 4 = Sakurai et al., 2005; 5 = Peyron et al., 1998; 6 = Elias et al., 1998). ACN, amygdaloïd cortical nucleus; AON, anterior olfactory nucleus; ARC, arcuate nucleus; DMH, dorso medial nucleus of the hypothalamus; EBOP, extra bulbar olfactory pathway; EC, entorhinal cortex; LH, lateral hypothalamus; NT, nervus terminalis; OB, olfactory bulb; OE, olfactory epithelium; OT, olfactory tubercle; Pir, cortex piriform; POA, preoptic area; PVN, paraventricular nucleus of the hypothalamus; TT, tenia tecta; VMH, ventro medial nucleus of the hypothalamus.

At the central level, many hormones and neuropeptides such as vasoactive intestinal peptide (VIP, Garcia-Llanes et al., 2003), cholecystokinin (CCK, Tanganelli et al., 2001), NPY (Matsutani et al., 1988), LHRH/GnRH (Apelbaum et al., 2005), and insulin (Fadool et al., 2011) may modulate olfactory processing. However, besides such hormonal mechanisms, a body of data (Doucette et al., 2007; Doucette and Restrepo, 2008; Fletcher and Chen, 2010) suggests the existence of a neuronal centrifugal modulation of olfactory bulb (OB) activity in different tasks, including the modulation of olfactory sensitivity toward food odorants (Pager et al., 1972; Pager, 1978; Royet et al., 1983). Different pathways (Figure 1), which are known to connect hypothalamic nuclei with olfactory centers, could be the neuroanatomical substrates accounting for the observations reported by Pager (1978). Among these, the “orexin/hcrt neurones” appear to be a likely candidate. Indeed, orexin/hypocretin (hcrt), a neuropeptide involved in sleep/wake regulation (Sakurai et al., 1998, 2010; Sakurai and Mieda, 2011), is also involved in feeding behavior (Horvath and Gao, 2005). Many studies have demonstrated that it could act by modulating olfactory sensitivity according to satiety (Aimé et al., 2007; Julliard et al., 2007; Prud’homme et al., 2009, for review: Palouzier-Paulignan et al., 2012). The cytological, connectional (Hahn and Swanson, 2010, 2012), immunohistological, and molecular (Peyron et al., 1998; Sakurai et al., 2005; Swanson et al., 2005) characterization of the lateral hypothalamus (LH) has already been performed. These studies suggest the existence of a functional loop between olfactory centers and the hypothalamus, which could explain the modulation of olfaction depending on energy balance. Unfortunately, inputs that project to orexin neurons have been shown in mice (Sakurai et al., 2005), while the LH orexin neuron projections to the OB have been shown in rats (Peyron et al., 1998; Nambu et al., 1999). Showing the existence of synaptic output from LH orexin neurons to the OB is a prerequisite to validate the reality of this loop in mice. Owing to the fact that the orexin immunostaining pattern presents variations amongst rodents, for instance between rats and hamsters (Nixon and Smale, 2007), the first preliminary step toward the validation of the putative loop between the LH and the OB was to validate data in mice obtained previously in rats (Peyron et al., 1998; Nambu et al., 1999). The aim of this study being established, future studies will address the question of connectivity through a trans-synaptic tracer experiment in order to draw the exact circuitry between OB and hypothalamus.

## Materials and Methods

### Animals

C57 mice were raised at the Cajal Institute mouse-breeding facility. All procedures followed the guidelines for animal care of the European Community Council (86/609/CEE), and were approved by the Bioethical Committee of the Spanish National Research Council (CSIC). The animals were kept under a constant 12–12 h dark-light cycle (light on at 07:00am). The day the vaginal plug was detected was designated as embryonic day 0 (E0). The mice were killed by hypothermia (embryos and P3) or with i.p. equithesin at a sub-lethal dose (3 mL/kg body weight; from P3 to adult) systematically at the same time of the diurnal cycle (04:00pm).

### Immunohistochemistry

After anesthesia, the mice were transcardially perfused with 4% paraformaldehyde (PF) in 0.1 M phosphate buffer (PB, pH 7.2). The brains were then post-fixed in PF for 2 h at 4°C, and coronal vibratome sections were obtained at 70 μm. One out of three sections was used for immunocytochemistry. Unspecific binding of antibodies was blocked by three washes in NGS 10% in Tris buffered saline with 5% Tween (TBS-T), followed by overnight incubation using primary rabbit anti-orexin A (Millipore AB3098; Sakurai et al., 1998) and diluted to 1:1000 in the buffer. Antibody binding was detected with the secondary antibody goat Anti-rabbit Alexa 488 (Invitrogen A11001) diluted to 1:700 during 2 h at room temperature. Sections were then washed three times in TBS-T. Nuclei were counterstained with Hoechst (1 μg/ml, Sigma-Aldrich Co.). The control consisted of substituting the primary antibody by an orexin B antibody, followed by the same immunohistological protocol just described.

### Microscopy and image processing

Optical sections were acquired using a Leica SP5 confocal microscope, 10× objectives HPC PL Apo CS dry UV, at a resolution of 1024 × 1024, without zoom magnification. The voxel size was 1.51 × 1.51 × 5.32. Whole coronal sections of the brains were reconstructed by doing XY mosaics using Leica software. For each XY position, six to seven optical sections were acquired in Z. Projections were made with a “Max intensity” algorithm using FIJI (an ImageJ plug-in). Relative quantification of orexin fibers was determined after thresholding the image in order to differentiate the neurite profile from the background. The measurements give values in percentage of surface area, thus expressing the density of orexin fibers per unit of area.

### Tissue dissection and RNA extraction and processing

After decapitation, embryo (E17) and newborn (P3) brains were rapidly dissected on ice. For adult males and 11-day-old mice (P11) the hypothalamus and cerebellum were quickly microdissected on ice systematically at the same time of the diurnal cycle (between 02:00 and 04:00pm). Liver tissue was also isolated from each animal. All of the collected tissues were immediately frozen in liquid nitrogen and stored at −80°C. Total RNA was extracted using the RNeasy Mini Kit (Qiagen). Possible DNA contaminants in the RNA preparation were eliminated by treatment with DNAse I (Qiagen). RNA quality was assessed with the Experion electrophoresis system (Bio-Rad) and the Experion RNA StdSens Analysis Kit (Bio-Rad). Total RNA concentration was determined using the Nanodrop spectrophotometer (Thermo Scientific).

### Real-time PCR analysis

Reverse transcription was performed with 500 ng of total RNAs, using the High-Capacity RNA-to-cDNA Master Mix (Applied Biosystem). For qPCR, the Fast SYBR Green Master Mix (Applied Biosystems) was used. Each reaction contained 1 μl of cDNA diluted 1:10 and 200 nM of gene specific intron-spanning primers. The sequences of the primers used for *prepro-orexin (Ppox)* amplification were 5′-GGCACCATGAACTTTCCTTC-3′ and 5′-GACAGCAGTCGGGCAGAG-3′. TATA box binding protein (Tbp) RNA expression was used as an endogenous control. The sequences of the primers were 5′-GGGAGAATCATGGACCAGAA-3′ and 5′-CCGTAAGGCATCATTGGACT-3′. Real-time PCR reactions were performed on a Step-One Plus thermocycler (Applied Biosystems). PCR conditions were 20 s at 95°C, followed by 40 cycles of 3 s at 95°C and 30 s at 60°C. Relative quantitation of gene expression (RQ) was based on the ΔΔ*C*_t_ method.

## Results

### Orexin in adult mice

We analyzed orexin A staining all along the antero-posterior axis of the mouse brains by immunohistochemistry. We selected confocal coronal sections, from the OB to the cerebellum, to perform a semi-quantitative evaluation of orexin A staining (Table 1), and compared the results with rat orexin staining (Peyron et al., 1998). For each brain area reported in Table 1, we quantified the percentage of orexin fibers (see “Materials and Methods”). Our data revealed that in mice, the orexin A immunostaining was as follows:(i) the localization of orexin A neuronal cell bodies in the LH (ii) a wide distribution of orexin A fibers in all brain areas except the cerebellum and caudate putamen, (iii) a strong presence of orexin A fibers in the thalamus and the hypothalamus. Therefore, the mouse orexin A pattern is similar to that in rats, even though there are some minor differences.

**Table 1:** **Semi-quantitative estimation of the density of presence in different brain areas**.

AMYGDALE		
AHiAL	Amygdalohippocampal area, anterolateral part	0.01 < *X* < 0.49
BLA	Basolateral amygdaloid nucleus, anterior part	0.01 < *X* < 0.49
BLP	Basolateral amygdaloid nucleus, posterior part	0.01 < *X* < 0.49
BLV	Basolateral amygdaloid nucleus, ventral part	0.01 < *X* < 0.49
BMA	Basomedial amygdaloid nucleus, anterior part	0.49 < *X* < 0.99
BMP	Basomedial amygdaloid nucleus, posterior part	0.01 < *X* < 0.49
ASt	Amygdalostriatal transition area	0.01 < *X* < 0.49
CeC	Central amygdaloid nucleus, capsular part	0.49 < *X* < 0.99
CeL	Central amygdaloid nucleus, lateral division	0.49 < *X* < 0.99
LaVL	Lateral amygdaloid nucleus, ventrolateral part	0.01 < *X* < 0.49
LaVM	Lateral amygdaloid nucleus, ventromedial part	0.01 < *X* < 0.49
MeAD	Medial amygdaloid nucleus, anterodorsal	*X* > 1
MeAV	Medial amygdaloid nucleus, anteroventral part	*X* > 1
MePD	Medial amygdaloid nucleus, posterodorsal part	0.01 < *X* < 0.49
MePV	Medial amygdaloid nucleus, posteroventral part	0.01 < *X* < 0.49
PLCo	Posterolateral cortical amygdaloid area	0.01 < *X* < 0.49
PMCo	Posteromedial cortical amygdaloid area	0.01 < *X* < 0.49
**BASAL GANGLIA**		
MS	Medial septal nucleus	0.01 < *X* < 0.49
**CORTEX**		
AIP	Agranular insular cortex, posterior part	0.01 < *X* < 0.49
Au1	Primary auditory cortex	0.01 < *X* < 0.49
AuD	Secondary auditory cortex, dorsal area	0.01 < *X* < 0.49
AuV	Secondary auditory cortex, ventral area	0.01 < *X* < 0.49
Cg1	Cingulate cortex, area 1	0.49 < *X* < 0.99
Cg2	Cingulate cortex, area 2	0.49 < *X* < 0.99
CxA	Cortex-amygdala transition zone	0.49 < *X* < 0.99
DEn	Dorsal endopiriform claustrum	0.01 < *X* < 0.49
Dl	Dysgranular insular cortex	0.01 < *X* < 0.49
DIEnt	Dorsal intermediate entorhinal cortex	0.01 < *X* < 0.49
DLEnt	Dorsolateral entorhinal cortex	0.01 < *X* < 0.49
DP	Dorsal peduncular cortex	0.49 < *X* < 0.99
Ect	Ectorhinal cortex	0.01 < *X* < 0.49
LO	Lat orbital cortex	0.49 < *X* < 0.99
MO	Medial orbital cortex	0.49 < *X* < 0.99
Ml	Primary motor cortex	0.01 < *X* < 0.49
M2	Secondary motor cortex	0.01 < *X* < 0.49
PRh	Perirhinal cortex	0.01 < *X* < 0.49
PRh	Perirhinal cortex	0.01 < *X* < 0.49
S1BF	Primary somatosensory cortex, barrel field	0.01 < *X* < 0.49
S1DZ	Primary somatosensory cortex, dysgranular zone	0.01 < *X* < 0.49
S1FL	Primary somatosensory cortex, forelimb region	0.01 < *X* < 0.49
S1HL	Primary somatosensory cortex, hindlimb region	0.01 < *X* < 0.49
S1ULp	Primary somatosensory cortex, upper lip region	0.01 < *X* < 0.49
S2	Secondary somatosensory cortex	0.01 < *X* < 0.49
TeA	Temporal association cortex	0.01 < *X* < 0.49
V2L	Secondary visual cortex, lateral area	0.01 < *X* < 0.49
VEn	Ventral endopiriform claustrum	0.49 < *X* < 0.99
VO	Ventral orbital cortex	0.49 < *X* < 0.99
**HYPOCAMPE**		
CA1	Field ca1 of the hippocampus	0.01 < *X* < 0.49
CA2	Field ca2 of the hippocampus	0.01 < *X* < 0.49
CA3	Field ca3 of the hippocampus	*X* = 0
DG	Dentate gyrus	*X* = 0
**HYPOTHALAMUS**		
AHA	Anterior hypothalamic area, posterior part	*X* > 1
Arc	Arcuate hypothalamic nucleus	*X* > 1
DA	Dorsal hypothalamic area	*X* > 1
DM	Dorsomedial hypothalamic nucleus	*X* > 1
LA	Lateroanterior hypothalamic nucleus	0.49 < *X* < 0.99
LH	Lateral hypothalamic area	*X* > 1
MPA	Medial preoptic area	*X* > 1
PaAP	Paraventricular hypothalamic nucleus, anterior parvic	*X* > 1
PaLM	Paraventricular hypothalamic nucleus, lateral magnoce	*X* > 1
PaV	Paraventricular hypothalamic nucleus, ventral part	*X* > 1
Pe	Periventricular hypothalamic nucleus	0.01 < *X* < 0.49
PH	Posterior hypothalamic nucleus	0.01 < *X* < 0.49
PMV	Premammillary nucleus, ventral part	*X* > 1
Te	Terete hypothalamic nucleus	0.49 < *X* < 0.99
VMH	Ventromedial hypothalamic nucleus	*X* > 1
SCH	Supra chiasmatic nucleus	*X* = 0
**MIDBRAIN/HINDBRAIN**		
APTD	Anterior pretectal nucleus, dorsal part	0.49 < *X* < 0.99
InC	Interstitial nucleus of Cajal	*X* > 1
IP	Interpeduncular nucleus	*X* > 1
IF	Interfascicular nuclei	*X* > 1
SN	Subs. Nigra	0.49 < *X* < 0.99
**OLFACTORY SYSTEM**		
AOB	Accessory olfactory bulb	0.01 < *X* < 0.49
AON	Anterior olfactory nuclei	0.49 < *X* < 0.99
TT	Dorsal tenia tecta	0.49 < *X* < 0.99
CoPyr	Pyriform cortex	0.49 < *X* < 0.99
MOB	Main olfactory bulb	0.01 < *X* < 0.49
**SEPTUM**		
**Ts**	Triangular septum nuclei	0.49 < *X* < 0.99
**STRIATUM**		
AA	Anterior amygdaloid area	0.49 < *X* < 0.99
ACo	Anterior cortical amygdaloid area	0.49 < *X* < 0.99
CPu	Caudate putamen (striatum)	0.01 < *X* < 0.49
ICjM	Island of Calleja, major island	0.49 < *X* < 0.99
LSD	Lateral septal nucleus, dorsal part	0.49 < *X* < 0.99
LSI	Lateral septal nucleus, intermediate part	0.49 < *X* < 0.99
**TECTUM**		
DpG	Deep gray layer of the superior colliculus	
DpWh	Deep white layer of the superior colliculus	
**THALAMUS**		
AD	Anterodorsal thalamic nucleus	*X* = 0
APTD	Anterior Pretectal nucleus, Dorsal	*X* = 0
CM	Central medial thalamic nucleus	*X* > 1
DLG	Dorsal lateral geniculate nuclei	*X* = 0
Gus	Gustatory thalamus nuclei	0.01 < *X* < 0.49
IGL	Intra geniculate leaf	*X* > 1
LDDM	Laterodorsal thalamic nucleus, dorsomedial part	0.01 < *X* < 0.49
LDVL	Laterodorsal thalamic nucleus, ventrolateral part	0.01 < *X* < 0.49
LHbL	Lateral habenular nucleus, lateral part	*X* = 0
LHbM	Lateral habenular nucleus, medial part	*X* = 0
MD	Mediodorsal thalamic nucleus	*X* > 1
MDM	Mediodorsal thalamic nucleus, medial part	0.01 < *X* < 0.49
OPT	Olivary pretectal nucleus	*X* = 0
PAG	Thalamus gray	*X* > 1
PC	Paracentral thalamic nucleus	*X* > 1
PF	Parafacicular thalamus nuclei	*X* > 1
Po	Post thalamus nuclei	0.01 < *X* < 0.49
PT	Paratenial thalamic nucleus	*X* > 1
PV	Paraventricular thalamic nucleus	*X* > 1
PVA	Paraventricular thalamic nucleus, anterior part	*X* > 1
PVP	Paraventricular thalamic nucleus, posterior part	*X* > 1
Re	Reuniens thalamic nucleus	*X* > 1
sm	Stria medullaris	*X* > 1
VLGMC	Vent lat genic magn	0.49 < *X* < 0.99
VLGPC	Vent lat genic magn	0.49 < *X* < 0.99
VL	Ventro lateral thalamus	*X* = 0
VPL	Ventro-postero-lateral thalamus	*X* = 0
VPM	Ventro postero medial thalamus	*X* = 0
SCO	Sub commissural organ	0.49 < *X* < 0.99
SI	Substancia inominata	0.49 < *X* < 0.99
cp	Cereb pedunc basal pt	*X* > 1

**Figure 2:**
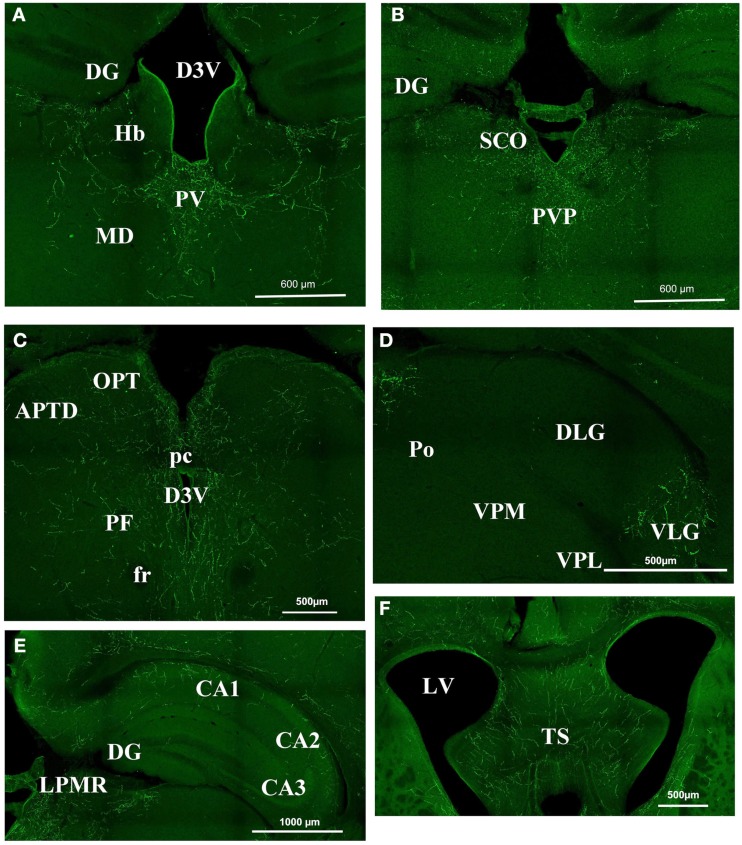
**Orexin staining in the thalamus, hippocampus, and septum**. **(A)** Periventricular area Bregma −1.82. Strong staining is visible at the level of the PV, while there is no staining in the DG, the Hb, and the MD. **(B)** Periventricular area at Bregma −2.30. Note the strong staining in the PVP and SCO and still the absence of staining in the DG. **(C)** Periventricular area at Bregma −2.80. There is strong staining around the third ventricle, but staining is faint in the OPT and the APTD. **(D)** Geniculate nucleus. Orexin is present in the VLG but absentin the DLG, the Po, the VPM, and VPL. **(E)** Hippocampal structures at Bregma −2.3. Note the staining in CA1 CA2 but not in CA3. **(F)** Strong staining in the TS. APTD, anterior pretectal nucleus dors.; D3V, dorsal third ventricle; DG, dentate gyrus; DLG, dorsal lateral geniculate; fr, fasciculus retroflexus; Hb, habenular nucleus; LPMR, lat. post. thal. nu. LV, lateral ventricle; MD, medio dorsal thalamus nucleus; OPT, olivary pretectal nucleus; pc, post commissure; PF, para fascicular thalamus nucleus; Po, post. thal. nu.; PV, paraventricular thalamic nucleus; PVP, paraventricular thalamic nucleus post; SCO, sub commissural organ; TS, triangular septal nucleus; VLG, ventral lateral geniculate; VPL, ventro postero lateral thal; VPM, ventro postero med. thal.

**Figure 3:**
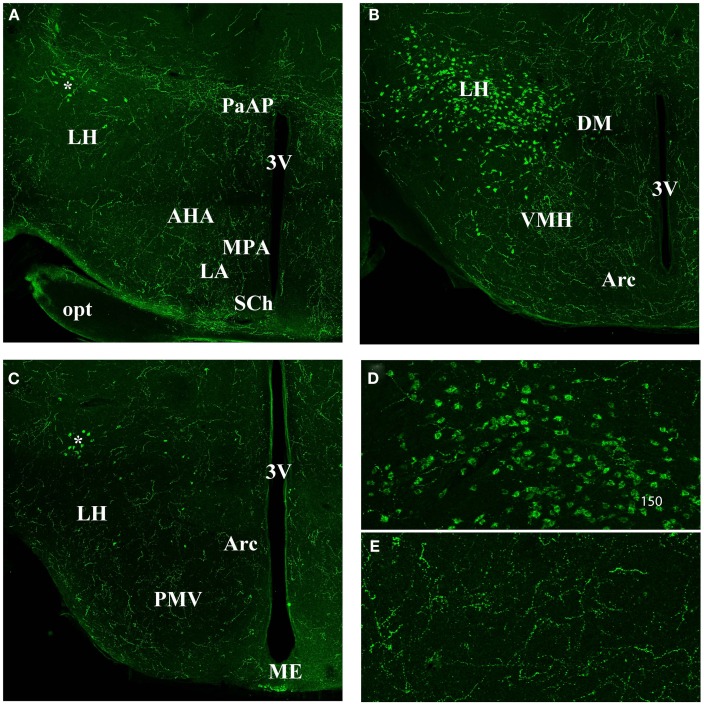
**Orexin staining in hypothalamic area (A) anterior part**. Note the strong staining in the PaAP, few orexin cell bodies (*) in the anterior LH, and general high staining in the LH, AHA, MPA, LA. Note the absence of staining in the SCh. **(B)** Orexin staining in the hypothalamus at the level of the orexin neuron population. Note the staining in the DM, VMH, and Arc nucleus. **(C)** Hypothalamus at the Posterior LH level. Note the few orexin positive neurons (*). Note general staining in the whole hypothalamus including LH, PMV, Arc, and Me. **(D)** Magnification of the orexin neuron cell bodies. **(E)** Magnification of orexin fibers in the hypothalamus. 3V, third ventricle; AHA, anterior hypothal. area; Arc, arcuate nucleus; DM, dorso medi hypothal. nu.; LA, lateroant. hypothal. nu.; LH, lateral hypothalamus; MPA, med. preoptic area nu.; ME, med. amyg. nu.; opt, optical nerve. PaAP, pa. anterior parvicell. pt.; PMV, premammill. nu. ventral; SCh, suprachiasmatic nu.; VMH, ventral hypothalamic nu.

In the mouse, the **thalamic area** appeared to be heavily orexin A labeled (Figure 2) at the level of the parafascicular thalamic nucleus (PF; Figure 2C), the paraventricular nuclei (PV; Figures 2A and 3B), the subcommissural organ (SCO; Figure 2B), the central medial, and mediodorsal (MD) thalamic nuclei (Figure 2A). Nevertheless, the immunostaining was not homogeneous throughout the whole thalamus. It appeared faintly stained in epithalamic areas: such as the lateral and medial habenular nucleus (Hb, Figure 2A), the olivary pretectal nucleus (OPT, Figure 2C) and the antero-pretectal-dorsal nucleus (APTD, Figure 2C). At the geniculate nucleus level, there was no orexin A in the dorso-lateral geniculate nucleus (DLG; Figure 2D), while the ventral lateral geniculate nucleus (VLG) was strongly stained (Figure 2D). There was almost no staining in the ventro lateral thalamus, the ventro-postero-lateral (VPL) and ventro-postero-medial thalamic nuclei (VPM; Figure 2D).

At **hippocampus level**, the staining was discrete and mostly located in CA1 and CA2. CA3 was consistently less stained than CA1 or CA2 (Figure 2E). The dentate gyrus was poorly labeled by orexin A staining (Figures 2A–C). The triangular septal nucleus (TS) area of the **septum** appeared to be stained consistently (Figure 2F).

The orexin A fibers were most abundant in **the hypothalamus** (Figure 3). The whole paraventricular area appeared to be very rich in orexin A fibers. In the anterior part of the hypothalamus (Figure 3A) the following parts were densely stained: the latero anterior hypothalamic nucleus (AHA); the medial preoptic area (MPA); the paraventricular anterior parvicellular part (PaAP) of the hypothalamic nucleus and the anterior LH. In contrast, no labeling was observed in both suprachiasmatic (SCh; Figure 3A) and supraoptic nuclei (not shown). In the LH, there is the orexin A neuron cell body population (Figure 3B). The dorso medial hypothalamus (DM), the ventral medial hypothalamus nucleus (VMH), and the arcuate nucleus (Arc) were densely stained. Caudally, the LH, the VMH, and the arcuate nucleus were stained (Figure 3C). Figures 3D,E present magnifications of the orexin A neuronal cell bodies and orexin A fibers respectively.

**Figure 4:**
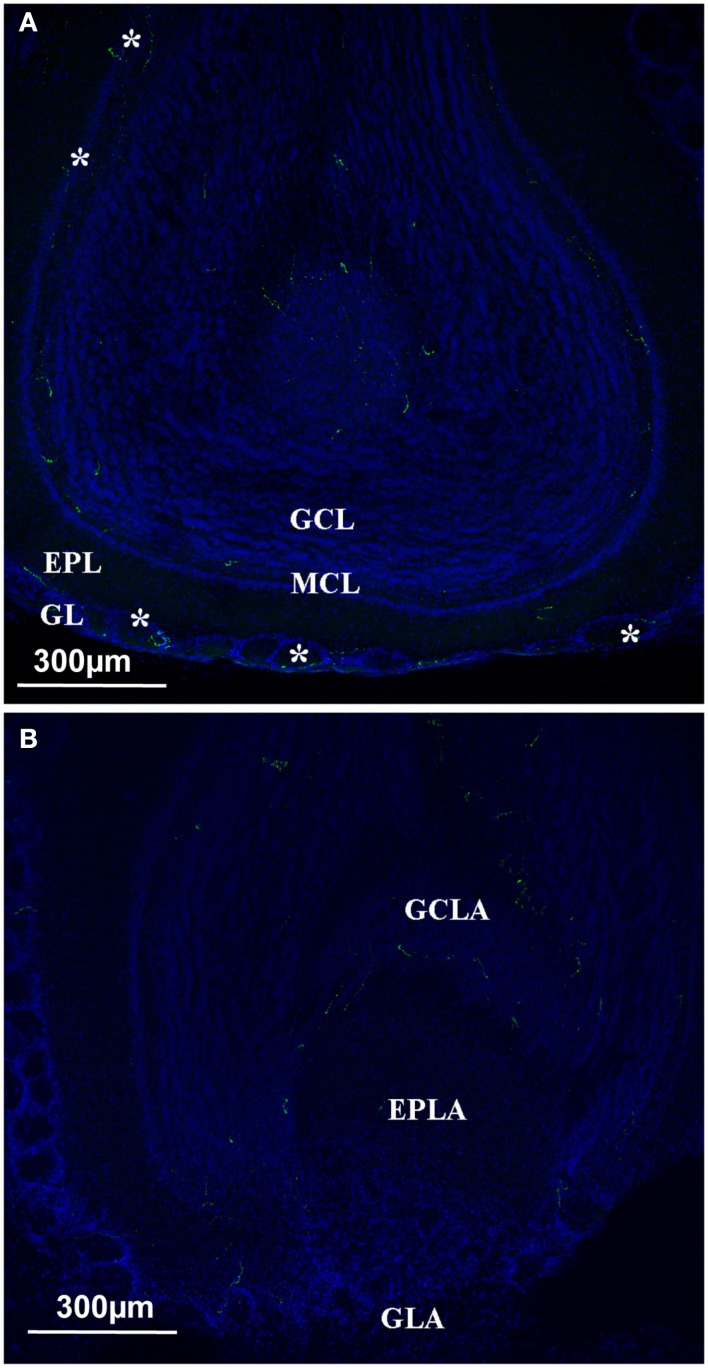
**Orexin staining at the level of the olfactory bulb**. **(A)** Main olfactory bulb; the orexin profile (green and white star) could be observed in all the different layers, including the Mitral cell layer arrow). **(B)** Orexin staining at the level of the Accessory Olfactory bulb. No staining at the level of the AOB except the GCLA. EPL, external plexiform layer; EPLA, external plexiform layer of the accessory olfactory bulb; GL, glomerular layer; GCL, granular cell layer; GCLA, granular cell layer of the accessory olfactory bulb; GLA, glomerular layer of the accessory olfactory bulb; MCL, mitral cell layer.

In the **OB**, there were fewer orexin A fibers than in the hypothalamic or thalamic areas; nonetheless they appeared to be present (Figure 4). According to the literature, the mitral layer in rats contains no orexin A fibers (Peyron et al., 1998). By contrast, orexin A fibers were seen in all the different layers of the main olfactory bulb (MOB), including the glomerular and the mitral cell layers (Figure 4A). Unlike the MOB, the accessory olfactory bulb (AOB) seemed to be devoid of orexin A fibers except in the granular cell layer (Figure 4B).

**Figure 5:**
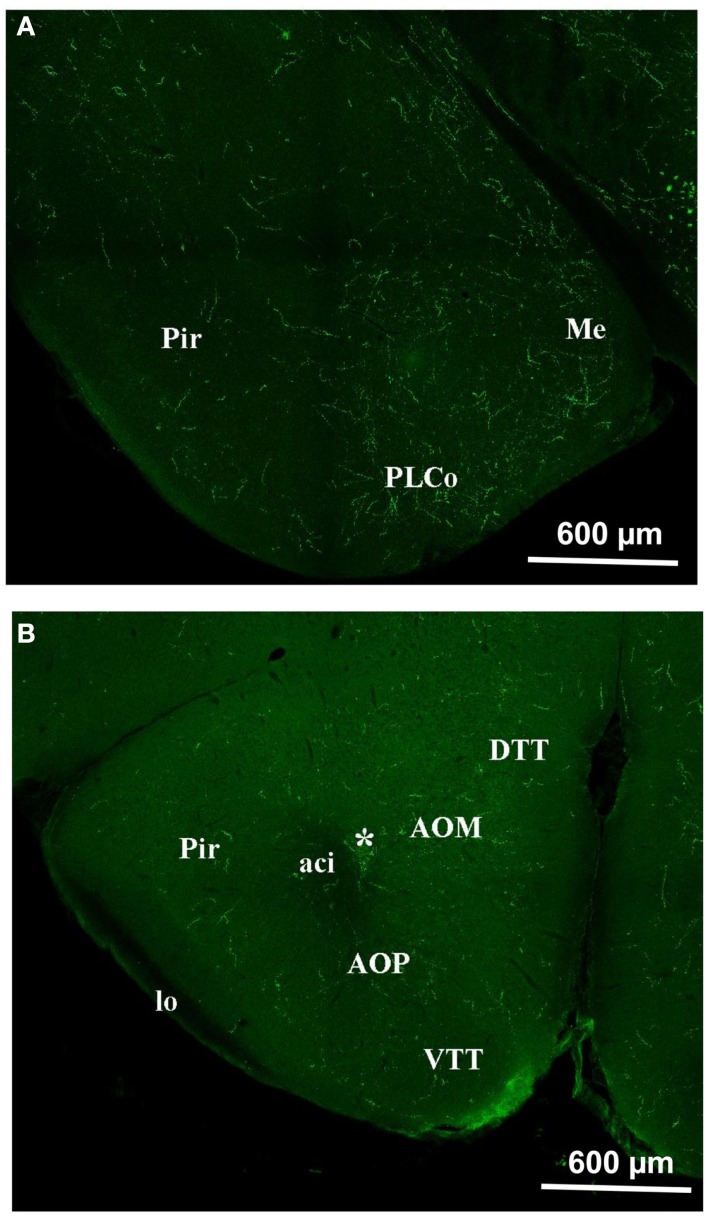
**Orexin staining in the olfactory cortex and the Amygdala**. **(A)** Piriform cortex and amygdala. Note the intense staining in the amygdala and also, though to a lesser extent, in the piriform cortex. **(B)** Olfactory cortex. Note the staining at the level of the DTT, VTT, AOM, AOP, Pir. *E/OV, Ependy/olfactory ventricle; aci, anterior commissure intrabulbar; AOM, anterior olfactory nucleus anterior; AOP, anterior olfactory nucleus posterior; DTT, dorsal tenia tecta; lo: lateral olfactory tract; Me, med amygdala; Pir, piriform cortex; PLCo, postlat cx amygdala nu.; VTT, ventral tenia tecta.

In the **olfactory cortex**, the density of orexin A appeared to lie between the MOB and the hypothalamus (Figure 5). Relatively dense staining was observed in the anterior olfactory nucleus (AOM and AOP), in the ventral and dorsal tenia tecta (VTT and DTT; Figure 5B). The same occurs in the piriform (Pir) and the entorhinal cortices (Figure 5A). We also investigated the amygdala, which is not olfactory *per se*, but is strongly connected to the olfactory system (Figure 5A). In areas of the amygdala, the staining was very dense as for instance in the medial amygdaloid nucleus (Me).

### Developmental expression of orexin

**Figure 6:**
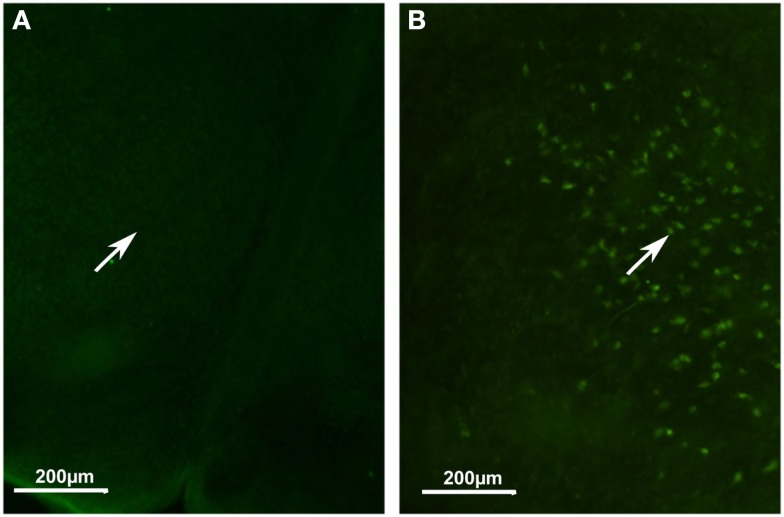
**Developmental expression of orexin A in mice**. **(A)** Hypothalamic area at P2. **(B)** Hypothalamic area at P11. Note orexin cell bodies only in **(B)** (white arrow).

**Figure 7:**
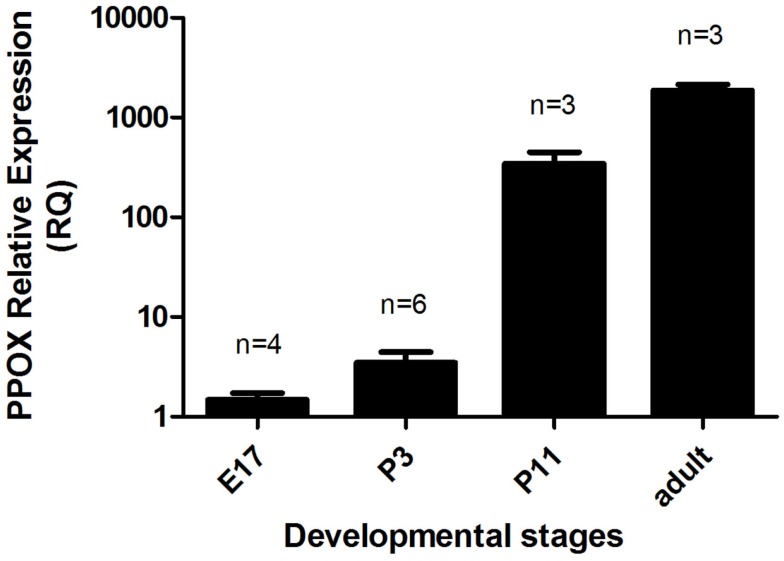
**Relative gene expression of Ppox in the brains of E17 and P3 mice, and in the hypothalamus of P11 and adult males**. Error bars represent the standard error. The number of samples per group is indicated above each column. The relative quantity of Ppox in each sample was normalized to the quantity of Tbp.

We also analyzed orexin A immunolocalization at different developmental stages, ranging from E11 to P11. We found the first evidence of the clear expression of orexin A at P11 (Figures 6A,B). At this stage, cell bodies in the LH appeared clearly stained even though just a few fibers were manifest. Orexin A and B are produced from a common polypeptide precursor, prepro-orexin *(Ppox)* by proteolytic processing (de Lecea et al., 1998; Sakurai et al., 1998). In order to confirm immunocytochemical data, we performed a real-time PCR analysis using *Ppox* primers (Figure 7). We showed that the level of expression of *Ppox* is barely detectable at E17 (RQ = 1.19) and very low at P3 (RQ = 4.17). In contrast, a strong increase in the level of expression was observed at P11 (RQ = 344.4) and in the adult stage (RQ = 1944.11). *Ppox* mRNA was undetectable in the liver at any developmental stage and in cerebellum of P11 and adult (data not shown).

## Discussion

Our data showed orexin A immunostaining in mice when compared with that described in rats. Orexin was found in nearly all brain areas except in the caudate putamen and cerebellum, with a strong expression in thalamus and hypothalamus. Moreover, while in the MOB was detected a sparse orexin A labeling throughout the different layers, almost no presence was detected in the AOB. The developmental expression of orexin A supports the hypothesis that orexin expression only appears post-natally.

### Adult orexin patterns

Even though convergent data revealed a link between olfactory perception and satiety (Pager et al., 1972; Pager, 1978; Yeomans, 2006), the neuroanatomical basis for this relationship and the exact connectome between olfaction centers and the hypothalamus is poorly known. Figure 1 summarizes the neuroanatomical connection between olfactory centers and the hypothalamus. From the peripheral olfactory system to the hypothalamus, two direct connections are described that do not synapse in the OB (Figure 1A). The first, the extra bulbar olfactory pathway (EBOP), is mainly known in lower vertebrates (fish, amphibians; Eisthen and Polese, 2006) and is composed of sensory neurons located in the OE, which project onto the preoptic area (POA) of the hypothalamus. The second, the NT, is a complex structure identified in many vertebrates including mammals and humans (Johnston, 1914; Fuller and Burger, 1990; Wirsig-Wiechmann, 1993, 1997). It is composed of ganglion(s) in which the neuronal cell bodies are located (Eisthen and Polese, 2006; Mousley et al., 2006; Kawai et al., 2009) that send branches towards both the OE and anterior POA of the hypothalamus. Due to its complex structure, the NT is not very well known. This is regrettable because the NT is certainly one of the systems most likely to be involved in the neuromodulation of the olfactory system, as it contains neuromodulatory peptides such as LHRH/GnRH and NPY neurons (Mousley et al., 2006; Kawai et al., 2009). However, both the EBOP and NT project to the POA, which is involved in the regulation of reproductive behavior rather than in food intake behavior, even though these two behaviors are inter-connected indirectly.

Besides, the system involving the NT, it has been possible to trace projections onto the LHRH/GnRH neurons localized in the POA, using Cre-loxP transgenic mice and/or pseudorabies virus infections (Boehm et al., 2005; Yoon et al., 2005). In Figure 1A, we present the projections from the olfactory and hypothalamic regions to the POA. Concerning the olfactory system, most olfactory cortex areas also appear to be connected to the OB and the OE. As for the hypothalamus connections, most of the hypothalamic nuclei appear to be connected to LHRH/GnRH neurons, among which are VMH, DMH, PVN, LH, and Arc. Therefore, LHRH/GnRH neurons, located in the POA, integrate many afferent signals; amongst which olfactory and hypothalamic inputs to regulate puberty onset, gametogenesis, estrus cycling, and sexual behavior (Gore, 2002) can be found. These connections appear to be bidirectional, since LHRH/GnRH appears to innervate the anterior piriform olfactory cortex (Boehm et al., 2005), indicating the possibility that LHRH/GnRH neurons could in turn modulate both olfactory/pheromonal processing. This has to be considered keeping in mind that both LHRH/GnRH and NPY in axolotl modulate and/or balance both sensitivity to food odorants and pheromones during reproduction periods (Mousley et al., 2006; Kawai et al., 2009).

In the context of olfactory system-hypothalamus connections (Price et al., 1991), the orexin network also has to be considered. Orexins are neuropeptides involved in sleep/waking regulation and food intake behavior (Sakurai et al., 1998, 2010; Aimé et al., 2007; Prud’homme et al., 2009). In rats, orexin neuron cell bodies are located in the perifornical nucleus, and in the dorsal and lateral hypothalamic areas (LHA; Peyron et al., 1998; Nambu et al., 1999). Interestingly, it has been suggested that differentiations in each LHA are involved in the control of specific behavior, with the involvement of the LHA suprafornical region in the control of food intake behavior (Hahn and Swanson, 2010, 2012). High resolution studies of the orexin neuron population in the LH have been carried out (Swanson et al., 2005) showing the extreme complexity of this brain area. In the present study, we focus on the orexin projection fibers – especially in the OB- rather than on the specific segregation of orexin cell bodies within the hypothalamus.

On the one hand, in mice, it has been demonstrated that orexin A neurons receive input from olfactory cortex areas, such as the tenia tecta (Figure 1B; Sakurai et al., 2005). On the other hand, in rats, it has been demonstrated that orexin neurons send projections to the olfactory cortex and OB (Figure 1B; Peyron et al., 1998). The aim of this work was to validate in mice the orexin projection patterns as described in rats (Peyron et al., 1998) with a special focus on the olfactory system.

Our present data show that the main orexin patterns in mice share many features with those in rats (Peyron et al., 1998; Nambu et al., 1999). The comparison of orexin A vs. orexin B staining attests the specificity of orexin A staining (Appendix). Extensive projections were present in nearly all brain areas except the cerebellum, and a high concentration of orexin projections was found in the paraventricular area of the hypothalamus and the thalamus. Amongst the differences to be noted is the absence of staining in the caudate putamen. Otherwise, there was only a small difference between rats and mice in terms of the relative level of the presence of orexin A fibers.

We then investigated in more detail the orexin projections at the level of the OB and olfactory cortex in mice. At the OB level, the density of orexin fibers was noticeably low. Nevertheless, none of the different layers were devoid of orexin fibers, and clear fibers could be seen in the mitral cell layer. At the level of the OB, the pattern of distribution appeared to be different in mice and rats, since in rats few orexin fibers were located in the glomerular and internal granular layers and none in the mitral cell layer (Peyron et al., 1998). To our knowledge, the AOB has not been investigated in rats. In mice, we found that the AOB was nearly devoid of orexin projections, except for the granular level of the AOB.

Our results show that the orexin A pattern is similar to those previously described in rats. Orexin fibers on the OB could have two origins. The first origin could be that fibers come from only one cell body population- those that only we and other researchers found to be located in the LH. The second origin could be that fibers, at least in the OB, originate from the olfactory mucosa, since some olfactory receptor neurons are orexin positive (Caillol et al., 2003). However, the second hypothesis seems to be ruled out since the olfactory neurons only project in the glomerular layer, and that we find orexin fibers in all the different layers of the OB. Thus, the projection of LH orexin neurons to the OB seems to be consensual. However, we cannot claim that synaptic connections of these orexin projections occur at the OB level. In order to establish the precise circuitry between LH and OB, the whole circuitry has to be investigated in detail using a trans-synaptic tracer strategy to demonstrate that LH orexin neurons constitute a true neuronal circuit between the OB and the LH.

### Developmental orexin patterns

Considering the developmental expression pattern, orexin has been reported in rats as early as E19 (Van der Pol et al., 2001; Steininger et al., 2004). However, this is highly controversial since other authors have not reported orexinergic neurons before post-natal day 15 (Yamamoto et al., 2000), or at best during the first post-natal week (Stoyanova et al., 2010). Our immunocytochemical results tend to support the late expression of orexin neurons, since we found in mice no evidence of orexin expression at P2, while at P11 instead we showed the presence of orexin neuronal cell bodies. Using qPCR, we observed that significant variations in *Ppox* mRNA reflect the synthesis of orexin peptides. Indeed, our qPCR results validate our immunohistological results, since at P2–3 the range of orexin expression is around 500 times lower than that in adults. This supports the idea that the beginning of orexin expression should be between P2–3 and P11. However, the low level of orexin fibers suggests that the full development of orexin neurons is not complete at P11 in mice.

## Conclusion

In this study we demonstrate that most of the characteristics of orexin in mice are similar to those reported in rats. Since it has been demonstrated in rats that orexin could modulate olfactory perception depending on the energy balance of the body, this work strongly supports the idea that this is also true in mice. However, trans-synaptic tracing experiments need to be done in order to clearly demonstrate connectivity between the two systems.

## Conflict of Interest Statement

The authors declare that the research was conducted in the absence of any commercial or financial relationships that could be construed as a potential conflict of interest.

## Figures and Tables

**Figure A1:**
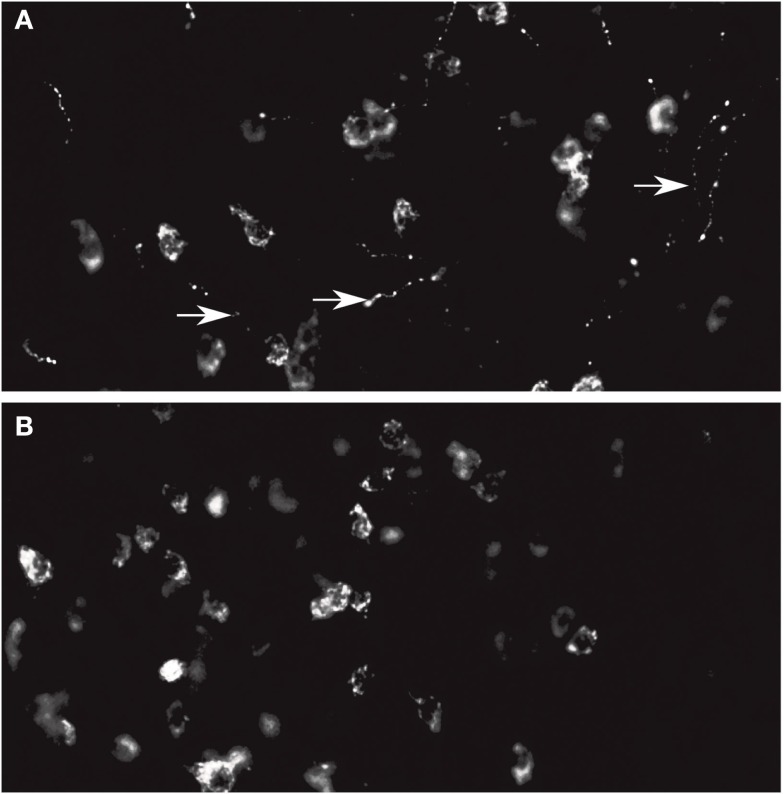
**(A)** Orexin A staining in the LH. Note the staining at the level of the cell bodies and fibers. **(B)** Orexin B staining in the LH. Note that the fibers are not stained. In this experiments only primary antibody is different.

## Abbreviations

3V, third ventricle; Aci, anterior commissure intrabulbar; AHA, anterior hypothal. area; AOB, accessory olfactory bulb; AOM, anterior olfactory nucleus anterior; AOP, anterior olfactory nucleus posterior; APTD, anterior pretectal nucleus dors.; Arc, arcuate nucleus; CCK, cholecystokinin; D3V, dorsal third ventricle; DG, dentate gyrus; DLG, dorsal lateral geniculate; DM, dorso medi hypothal. nu.; DTT, dorsal tenia tecta; E/OV, ependy/olfactory ventricle; EPl, external plexiform layer; fr, fasciculus retroflexus; Gl, glomerular level; GlA, glomerular layer of the accessory olfactory bulb; GnRH, gonadotropin-releasing hormone; GrA, granular cell layer of the accessory olfactory bulb; GrO, granular cell layer; Hb, habenular nucleus; Ipl, internal plexiform layer; LA, lateroant. hypothal. nu.; LH, lateral hypothalamus; LPMR, lat. post. thal. nu.; MD, medio dorsal thalamus nucleus; Me, med amygdala; ME, median eminence; Mi, mitral cell layer; MiA, mitral cell layer of the accessory olfactory bulb; MOB, main olfactory bulb; MPA, med. preoptic area nu.; NPY, neuropeptide Y; OE, olfactory epithelium; OB, olfactory bulb; OPT, olivary pretectal nucleus; opt, optical nerve; PaAP, pa. anterior parvicell. Pt. pc, post commissure; PF, para fascicular thalamus nucleus; Pir, piriform cortex; PLCo, postlat cx amygdale nu.; PMV, premammill. nu. ventral; Po, post. thal. nu.; PV, paraventricular thalamic nucleus; PVP, paraventricular thalamic nucleus post; SCh, suprachiasmatic nu.; SCO, sub commissural organ; SSS, sensory specific satiety; TS, triangular septal nucleus; VIP, vasoactive intestinal peptide; VLG, ventral lateral geniculate; VMH, ventral hypothalamic nu.; VPL, ventro postero lateral; VPM, ventro postero med. thal.; VTT, ventral tenia tecta.
